# Quality of life in women with breast cancer undergoing neoadjuvant chemotherapy: comparison between PICC and PICC-port

**DOI:** 10.1007/s12282-024-01608-z

**Published:** 2024-07-09

**Authors:** Fulvio Pinelli, Francesco Barbani, Barbara Defilippo, Angela Fundarò, Alessandra Nella, Valentina Selmi, Stefano Romagnoli, Gianluca Villa

**Affiliations:** 1 Vascular Access Center, Department of Anesthesia and Intensive Care, Careggi University Hospital, Largo Brambilla 3, 50134 Florence, Italy; 2 Department of Nursing, Careggi University Hospital, Florence, Italy; 3 Department of Health Sciences (DSS), Section of Anesthesiology, Intensive Care and Pain Medicine, University of Florence, Viale Pieraccini, 6, 50139 Florence, Italy; 4 Department of Health Sciences (DSS), University of Florence, Viale Pieraccini, 6, 50139 Florence, Italy

**Keywords:** Quality of life, Venous access, Peripherally inserted central venous catheter (PICC), PICC-ports, Complications

## Abstract

**Background:**

Peripherally inserted central catheters (PICCs) and new type of arm-port, the PICC-port, are currently used for neoadjuvant chemotherapy treatment in patients with breast cancer. We aimed to compare Quality of Life (QoL) of patients receiving one of these two devices investigating overall satisfaction, psychological impact, as well as the impact on professional, social and sport activities, and local discomfort.

**Methods:**

We did a prospective observational before–after study of PICCs versus PICC-ports. Adult (aged ≥ 18 years) females with breast cancer candidate to neoadjuvant chemotherapy were included. The primary outcome was QoL according to the Quality-of-Life Assessment Venous Device Catheters (QLAVD) questionnaire assessed 12 months after device implantation.

**Results:**

Between May 2019 and November 2020, of 278 individuals screened for eligibility, 210 were enrolled. PICC-ports were preferred over PICCs with a QLAVD score of 29 [25; 32] vs 31 [26; 36.5] (*p* = 0.014). Specifically, most QLAVD constructs related to psychological impact, social aspects, and discomfort were in favor of PICC-ports vs PICC, especially in women under the age of 60. Overall, pain scores at insertion and during therapy administration were not significantly different between the two groups, as well as infection, secondary malpositioning, thrombosis, or obstruction of the device.

**Conclusions:**

In women with breast cancer undergoing neoadjuvant chemotherapy, PICC-ports were overall better accepted than PICCs in terms of QoL, especially in those who were younger. Device-related complications were similar.

## Introduction

Breast cancer is the most frequent malignancy in women, accounting for 11.7% of all cancer cases, as reported in Global Cancer Statistics 2020, which was published by the International Agency for Research on Cancer [1]. Patients are frequently diagnosed at an early stage [2] and, in most of the cases, include relatively young and socially active individuals. Therefore, specific aspects should be considered in this population, such as psychological/relationship needs and family/working habits.

Women with early breast cancer are often candidate to neoadjuvant chemotherapy, a strategy aiming at reducing tumor size allowing a more conservative surgery or, in most advanced cases, to make surgically resectable an otherwise inoperable tumor [3]. A safe and reliable vascular access device (VAD) is an essential part of the chemotherapy (CT) in these patients [4]. In daily clinical practice, both peripheral inserted central catheters (PICCs) and totally implantable vascular access ports (TIVAPs) are currently used for CT treatment in patients with breast cancer [5, 6], and both carrying advantages and disadvantages.

The use of PICC as an alternative to traditional chest TIVAPs has increased over the last 2 decades, possibly due to several reasons, such as safety, ease of insertion and removal by nursing teams, and reduced waiting time [7]. On the other hand, PICCs have cosmetic disadvantages, due to the presence of an external visible part, and they may interfere with daily activities (e.g., bathing and showering) [8]. Furthermore, they need weekly medications, which may be inconvenient for patients and expensive for institutions. Finally, as external devices, they are at greater risk of dislodgement [9].

TIVAPs have the advantage of monthly or even bimonthly maintenance [10, 11], and they interfere less with daily activities such as bathing [8]. On the other hand, they are more invasive devices. In fact, TIVAPs have been traditionally implanted by accessing deep veins of the supraclavicular/infraclavicular area, carrying a risk of insertional immediate complications, such as hemorrhage and pneumothorax [12]. Furthermore, TIVAPs may also bring late onset complications, such as wound dehiscence and pocket infection [13]. Also, placing the reservoir into the anterior chest wall may raise concerns in terms of acceptance, especially in young female patients with breast cancer for cosmetic, psychological, and social reasons. An additional scar and a bulging body in the pectoral area may be troublesome in terms of body image. In fact, some patients may not want to make their illness public or may not want to remind themselves or their family members of their condition or even may experience it as a “social stigma” [14, 15].

In the last 2 decades, arm-ports have been considered in clinical practice to reduce the invasiveness of the procedure, decrease the risk of the intraoperative complications, and improve patients’ acceptance. Though, high rate of failure due to late complications (4–17%), most of them infective and thrombotic, has led to a residual use of arm-port in clinical practice [16–18].

Recently, with the aim of reducing these complications, an evolution of arm-ports, the PICC-ports, has been implemented into clinical practice. The consistent adoption of the state-of-the-art PICC insertion technique, namely: (a) the choice of a vein with appropriate catheter/vein ratio; (b) the systematic use of US-guided venipuncture; (c) the minimal trauma to the vein wall secondary to the adoption of micro-puncture kits; (d) the intra-procedural control of the catheter tip, mostly using intracavitary ECG, have led to a considerable improvement in terms of safety and reliability of these devices. PICC-port is a particular type of arm TIVAP, inserted according the current state-of-the-art of PICC insertion—ultrasound (US)-guided venipuncture of deep veins of the arm, micro-puncture kits, proper location of the tip preferably by intracavitary electrocardiogram (IC-ECG)—with placement of the reservoir at mid-arm [19]. Recently, PICC-ports have been advocated as an alternative to chest TIVAPs to reduce complications and improve patients’ acceptance. Clinical studies reported positive clinical outcomes with the use of these devices [19, 20]. Also, PICC-ports may be favorably accepted by female with breast cancer, a relatively young and socially active population [21]. In fact, avoiding the chest area or an external device, such as a PICC, may positively impact emotional well-being, relationships need, and family/working habits of these patients. Prioritizing quality of life (QoL) is critical in cancer care [22].

Although several studies have evaluated the quality of life (QoL) related to different devices in oncologic patients [15, 23], no studies compared PICC and PICC-port in terms of QoL and rate of complications in women undergoing neoadjuvant chemotherapy for breast cancer.

## Materials and methods

### Study design and setting

**Fig. 1:**
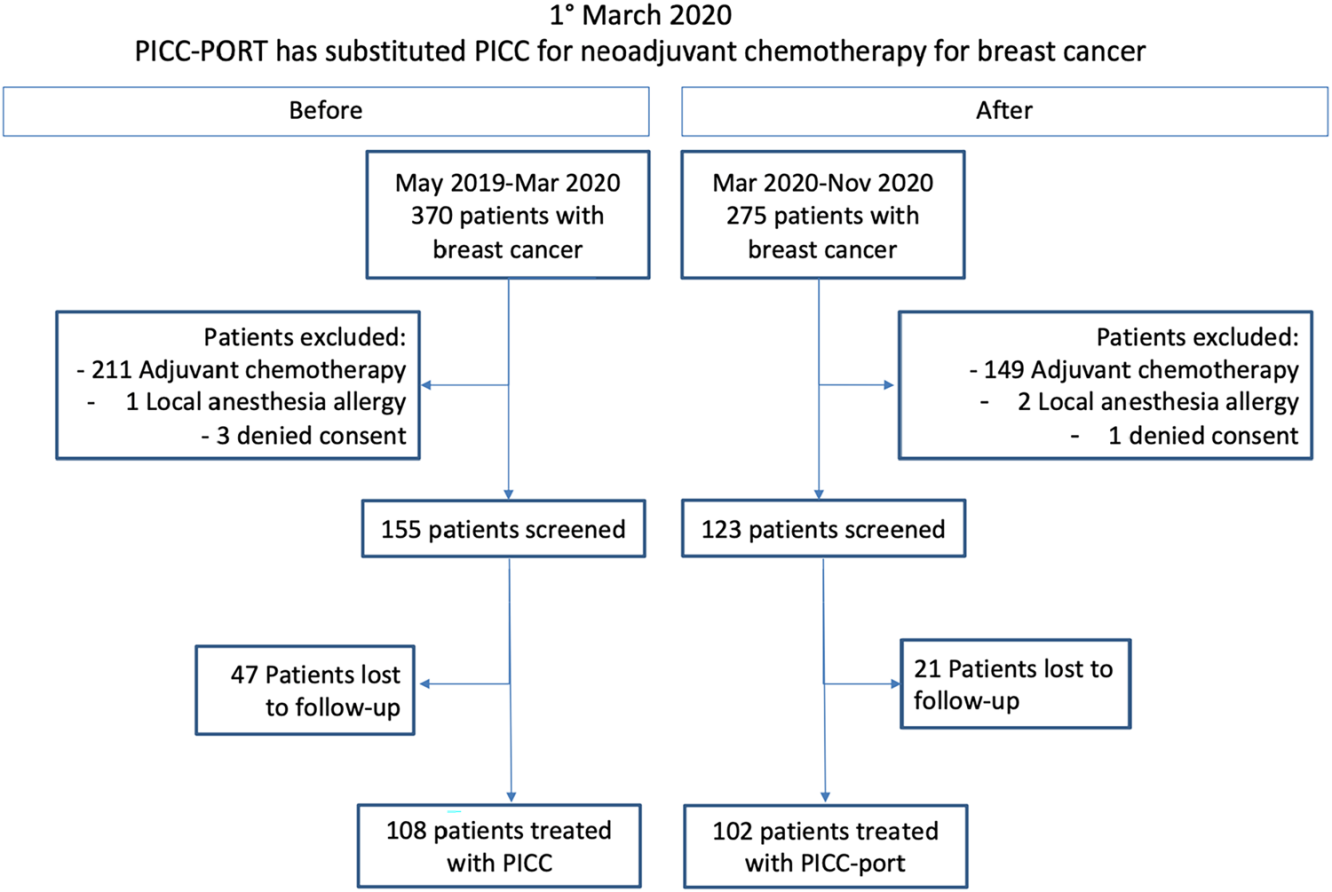
Inclusion and exclusion criteria, and selection process

A prospective observational before–after study was conducted at the Center of Vascular Access of the Department of Anesthesia and Intensive Care Medicine at Careggi University Hospital, Florence-Italy, between May 2019 and November 2020. All adult female patients with breast cancer with an indication for a VAD for neoadjuvant CT were considered eligible for this study. Inclusion and exclusion criteria, and the entire selection process are reported in Fig. 1.

### Type of vascular devices and implantation technique

In our center, VADs for CT are all implanted by expert vascular access specialists (anesthesiologists and nurses). PICCs were implanted as a standard of practice for breast cancer neoadjuvant CT until March 2020. From that time on, we have modified our practice, as we started implanting PICC-port for that indication.

The type of PICC implanted for this study was a single lumen Power PICC (Bard^®^, Becton & Dickinson, Franklin Lakes, NJ, USA). The type of PICC-port implanted were Perouse (Polysite^®^ 4000, Vygon, Ecouen, France) or Celsite Brachial (Port Celsite^®^ Baby/Brachial, B-Braun, Melsungen, Germany). Both PICCs and PICC-ports were in polyurethane and implanted according to our institution’s insertion protocols [24] which include: pre-procedural US vascular assessment following the RaPeVA protocol (RaPeVA = Rapid Peripheral Vein Assessment), measurement of vein diameter, and respect for a catheter/vein ratio less than or equal to 1:3, skin antisepsis with 2% chlorhexidine in 70% isopropyl alcohol, maximal barrier precautions, US-guided venipuncture using micro-introducer kit, use of intracavitary electrocardiography method to verify the correct position of the tip of the catheter at the cavo-atrial junction, location of the exit site/port reservoir in Dawson’s green zone (adopting tunneling from the yellow to the green zone, if necessary); for PICCs, catheter securement with adhesive stabilization systems and sealing of the exit site with cyanoacrylate glue; for PICC-ports, closing the wound with 4–0 intradermal absorbent sutures and application of cyanoacrylate glue. For PICCs, subsequent dressings and saline flushing were performed weekly, whereas for PICC-ports, saline flushing was provided after every administration of CT or, in any case, once a month. The care and maintenance of the devices was entrusted to specialized nurses of our Vascular Access Team, according to institutional protocols.

### Outcomes

The Quality-of-Life Assessment Venous Device Catheters (QLAVD) questionnaire is a test encompassing 30 questions exploring 7 aspects of life, with Likert or visual analogic scale answers was administered 12 months after VAD implantation to assess their quality of life. QLAVD was created after a validated and found reliable survey tool created by Marcy et al. [25], the QASSIC. QASSIC was translated into English from French, added with several questions, and finalized by Burbridge et al. [21] in a new survey tool, the QLAVD. Subsequently, Liu et al. [26] found that QLAVD had good content validity, had internal consistency, and had a high degree of reliability and stability. Aspects analyzed with QLAVD include pain during placement or use, cosmetic and privacy issues, the impact on professional, social and sport activities, and local discomfort. Procedural pain experienced by the patients was also recorded, as well as other negative experiences related with the implantation (e.g., tearing or tingling). Overall satisfaction score was the sum of each answer in addition to VAS pain scale at implantation. Therefore, the higher the score, the less the satisfaction.

As secondary outcomes, we explored early and late complications including: intra-procedural pain, infection, ecchymosis, hematoma, symptomatic catheter-related thrombosis (CRT), occlusion, and secondary malpositioning. We considered both local and catheter-related bloodstream infection (CRBSI).

### Definitions

Intraprocedural pain was defined as pain experienced during device implantation, and it was measured according to the Visuo-Analogic Scale (VAS). Local infection was defined by the presence of erythema and/or tenderness over the pocket of the reservoir/exit site and along the tunneled catheter to the vein access, with fever and regardless of the presence of purulent discharge. CRBSI was defined according to the Infectious Diseases Society of America guidelines [27]. Hematoma was defined as the collection of blood into the reservoir pocket (for PICC-port) or along the tunneled catheter (for PICC-port and PICC). Ecchymosis was defined as a discoloration of the skin, resulting from bleeding underneath, but without collection of blood. CRT was defined as the partial or complete thrombotic occlusion of the vessel as established by ultrasound venous scan (compression ultrasonography or color-Doppler), which was performed only when clinically suspected (tenderness, edema, and erythema of the arm). Occlusion was defined as the inability to infuse normal saline solution despite the manual pressure performed by a 10 ml syringe. Secondary malposition was defined as a tip migration from its initial position.

The local ethics committee approved the study, and all patients gave written informed consent (CEAVC #15,512). Patients’ records were anonymized and de-identified before the analysis.

### Statistical analysis

Quantitative variables were presented as mean (SD) or median [interquartile range] according to Shapiro–Wilk test for normality distribution, while qualitative one as absolute and relative frequencies.

To evaluate the difference in quantitative baseline clinical conditions and variable related to the VAD in the PICC and PICC-port groups, the Mann–Whitney test was used according to Shapiro–Wilk test for normality distribution, while for qualitative ones, the Chi-square or Fisher’s exact test were used according to expected absolute frequencies in each cross-table’s cell.

To assess the association between subject pain experience, treatment-related construct, overall satisfaction, psychological aspects, complications, and long-term outcomes with PICC/PICC-port group, a simple logistic regression model was used. OR and its 95% confidence interval were reported.

To evaluate possible difference in age groups (> 60 and ≤ 60 years) of association between PICC/PICC-port and belief that insertion of the device was a good thing to have done, degree of satisfaction with the device, easiness to present the device for treatment or blood sampling, fear of the device obstruction, and difficulties during local hygiene, a simple logistic regression model was used separately in the age subgroups. OR and its 95% confidence interval were reported.

## Results

One-thousand-one-hundred-and-thirty patients were screened in the considered period; among them, 210 women were enrolled and considered for the final analysis. The screening process is described in Fig. 1. Baseline characteristics of the two populations and variables related to the VAD insertion are described in Table 1. Patients well tolerated both the procedures (median overall VAS was 1 [0; 3] and 1.5 [0.5; 3] for PICC and PICC-port, respectively), with no significant differences between groups (*p* = 0.10). Venipuncture occurred on the contralateral arm with respect to the cancer site or, in case of bilateral tumors, in the patient's dominant limb.

**Table 1:** Patients’ baseline clinical conditions and variables related to the VAD implantation procedure

	Overall (*n* = 210)	PICC (*n* = 108)	PICC-port (*n* = 102)	*p* value
Patients’ baseline clinical conditions				
Age (years)	51 [44; 62]	53 [46; 64]	50 [43; 60]	0.05
BMI (kg/m^2^)	23 [21; 27]	24 [21; 27]	23 [21; 26]	0.30
Previous radiotherapy	8 (3.8%)	1 (0.9%)	7 (6.9%)	0.05
Previous chemotherapy	21 (10%)	8 (7.4%)	13 (12.8%)	0.05
Previous DVT	5 (2.4%)	5 (4.9%)	0 (0%)	0.04
Current antiplatelet drugs	13 (6.2%)	2 (1.9%)	11 (10.8%)	0.03
Current anticoagulation	10 (4.8%)	1 (0.9%)	9 (8.8%)	0.02
Current prophylactic LMWH	3 (1.4%)	1 (0.9%)	2 (2.0%)	0.03
Variables related to the VAD implantation procedure				
Attempts of venipuncture				0.0005
1	196 (93.3%)	107 (99.1%)	89 (87.3%)	
2	14 (6.7%)	1 (0.9%)	13 (12.8%)	
Type of vein				0.70
Brachial	81 (38.6.1%)	41 (38.0%)	40 (39.2%)	
Basilic	103 (49.0%)	51 (47.2%)	52 (51.0%)	
Axillary	26 (12.4%)	16 (14.8%)	10 (9.8%)	
Catheter diameter				0.001
4 Fr	59 (28.1%)	59 (54.6%)	0 (0%)	
4.5 Fr	21 (10%)	0 (0%)	21 (20.6%)	
5 Fr	130 (61.9%)	49 (45.4%)	81 (79.4%)	
Procedural time (min)	25 [20; 35]	20 [15; 25]	35 [30; 45]	0.001

*BMI* body mass index, *DVT* deep venous thrombosis, *LMWH* low-molecular-weight heparin

Patients in PICC group were explored for qualitative outcomes at 372 [345; 382] days, while those in the PICC-port group were explored at 355 [340; 379] days. Total QLAVD scored 29 [25; 33] in the overall population (PICC 31 [26; 36.5] vs PICC-port 29 [25; 32], *p* = 0.014). Patients’ quality of life described through the QLAVD is reported in Table 2 for both groups.

**Table 2:** Patients’ quality of life described through the Quality-of-Life Assessment Venous Device Catheters

		Overall (*n* = 210)	PICC (*n* = 108)	PICC-port (*n* = 102)	OR [95%CI]	*p* value	AUC [95% CI]
Subject pain experience							
Patients who experienced pain during device implantation (VAS ≥ 4)		17 (8.1%)	6 (5.6%)	11 (10.8%)	2.00 [0.72–5.56]	0.18	
Treatment-related construct							
Patients who experienced discomfort at the connection of infusion line (e.g., connection to PICC or needle insertion into PICC-port)	63 (30.0%)		30 (27.8%)	33 (32.4%)	1.25 [0.69–2.27]	0.50	
Patients who experienced discomfort during treatment infusion	4 (1.9%)		2 (1.9%)	2 (2.0%)	1.06 [0.18–6.25]	1.0	
Patients who experienced discomfort at the disconnection from the infusion line (e.g., disconnection to PICC or needle removal from PICC-port) after treatment	2 (1.0%)		1 (1.0%)	1 (1.0%)	1.06 [0.11–10.00]	1.0	
Overall satisfaction							
Patients who believed that insertion of the device was a good thing to have done		174 (82.9%)	82 (76.0%)	92 (90.2%)	2.86 [1.30–6.25]	0.01	0.63 [0.54–0.71]
Degree of satisfaction with the device (in a scale 0–10)		9.97 [9.97; 9.98]	9.97 [9.97; 9.98]	9.98 [9.96; 9.98]	2.22 [1.28–3.85]	0.005	0.60 [0.53–0.67]
Patients who would have another device if they had to have one implanted for another session of treatment during their life		190 (90.5%)	95 (88.0%)	95 (93.1%)	2.5 [0.77–10.00]	0.12	
Psychological aspects							
Patients who found the device easy to present for treatment or blood sampling		36 (17.1%)	26 (24.1%)	10 (9.8%)	0.35 [0.16–0.77]	0.01	0.63 [0.54–0.71]
Patients who felt the device was too visible		14 (6.7%)	7 (6.5%)	7 (6.9%)	1.06 [0.37–3.03]	0.91	
Patients who felt the device was unsightly or ugly		10 (4.8%)	3 (2.8%)	7 (6.9%)	2.38 [0.65–9.09]	0.20	
Patients who changed the way they dress due to the device		15 (7.1%)	10 (9.3%)	5 (4.9%)	0.53 [0.18–1.56]	0.25	
Patients who had people commented on the device when they see it		3 (1.4%)	1 (1.0%)	2 (2.0%)	1.79 [0.23–14.29]	0.58	
Patients who tried to cover the device with clothing		52 (24.8%)	37 (34.3%)	15 (14.7%)	0.33 [0.17–0.66]	0.01	0.63 [0.56–0.71]
Patients who felt anxious (worried) about the device		12 (5.7%)	9 (8.3%)	3 (2.9%)	0.37 [0.10–1.30]	0.12	
Patients who were worried about eventual damage of the device		7 (3.3%)	6 (5.6%)	1 (1.0%)	0.23 [0.04–1.43]	0.12	
Patients who was worried that the device might become blocked		26 (12.4%)	19 (17.6%)	7 (6.9%)	0.36 [0.15–0.88]	0.03	0.62 [0.53–0.72]
Patients who were bothered by the device during work-related activities		10 (4.8%)	8 (7.4%)	2 (2.0%)	0.29 [0.07–1.25]	0.10	
Patients who were bothered by the device when they shower, bathe, or perform personal hygiene		25 (11.9%)	23 (21.3%)	2 (2.0%)	0.09 [0.02–0.34]	0.001	0.73 [0.67–0.80]
Patients who were bothered by the device during sports or exercise		15 (7.1%)	10 (9.3%)	5 (4.9%)	0.53 [0.18–1.56]	0.25	
Patients who were bothered by the device during social activities		25 (11.9%)	23 (21.3%)	2 (2.0%)	0.09 [0.02–0.35]	0.001	0.73 [0.67–0.79]
Patients who were bothered by the device when they are lying down in bed		7 (3.3%)	6 (5.6%)	1 (1.0%)	0.23 [0.04–1.43]	0.10	
Patients who were hurt by the device		4 (1.9%)	2 (1.9%)	2 (2.0%)	1.06 [0.18–6.25]	0.10	
Patients who felt the device uncomfortable to touch		10 (4.8%)	8 (7.4%)	2 (2.0%)	0.29 [0.07–1.25]	0.10	

*VAS* Visuo-Analogic Scale

Most of the differences perceived by the patients in the PICC and in the PICC-port groups refer to self-determination, autonomy, social interactions, and preservation of body image. During the interviews, recurrent issues, mostly raised by younger patients, included: “work activities”, “my children need”, or “my femininity”. Older patients referred as major concerns mostly: “procedural pain”, “movement impairment”, or “difficulties in personal hygiene”. We thus have argued that the quality of life of patients with VAD should be evaluated along with patients’ expectancies associated with their age. Table 3 describes the effect that the variable “age” had on the qualitative outcomes, which significantly differed between groups.

**Table 3:** The effect of PICC/PICC-port on outcomes in age subgroups

	OR [CI 95%]	*p* value	AUC [CI 95%]
Belief that insertion of the device was a good thing to have done			
> 60 years	4.17 [0.66–25.00]	0.13	0.66 [0.53–0.79]
≤ 60 years	2.63 [1.10–6.25]	0.03	0.62 [0.52–0.72]
Degree of satisfaction with the device (in a scale 0–10)			
> 60 years	1.89 [0.67–5.26]	0.23	0.58 [0.46–0.70]
≤ 60 years	2.5 [1.28–5.03]	0.01	0.61 [0.54–0.69]
Easiness to present the device for treatment or blood sampling			
> 60 years	0.22 [0.05–0.97]	0.05	0.66 [0.55–0.78]
≤ 60 years	0.37 [0.16–0.89]	0.02	0.62 [0.52–0.73]
Fear of the device obstruction			
> 60 years	0.48 [0.10–2.27]	0.36	0.59 [0.43–0.75]
≤ 60 years	0.34 [0.12–0.99]	0.05	0.64 [0.52–0.76]
Difficulties during local hygiene			
> 60 years	0.11 [0.03–0.41]	0.001	0.72 [0.65–0.79]
≤ 60 years	0.12 [0.03–0.50]	0.01	0.73 [0.64–0.82]

OR > 1 demonstrates a positive effect of PICC-port on considered outcomes. OR < 1 demonstrates negative effect of PICC-port on considered outcomes; thus, in these cases, a PICC resulted in a greater acceptance

Complications and long-term outcomes related to the VAD insertion and management are reported in Table 4. Long-term outcomes (including catheter dislodgment, venous thrombosis, and catheter obstruction) were assessed at the patient’s interview.

**Table 4:** Complications and patients’ long-term outcomes

	Overall (*n* = 210)	PICC (*n* = 108)	PICC-port (*n* = 102)	OR [95% CI]	*p* value
Intraprocedural pain (VAS)	1.0 [0; 3]	1.0 [0; 3]	1.5 [0.5; 3]	1 [0.51–2.22]	0.10
Difficult tip-navigation	3 (1.4%)	0 (0%)	3 (2.9%)	3.28 [0.34–32.12]	0.28
Arterial puncture	0 (0%)	0 (0%)	0 (0%)		
Infection	2 (1.0%)	0 (0%)	2 (2.0%)	5.56 [0.25–99.50]	0.28
Ecchymosis	12 (5.7%)	1 (1.0%)	11 (10.8%)	9.09 [1.61–49.50]	0.01
Secondary malpositioning	6 (2.9%)	4 (3.7%)	2 (2.0%)	0.58 [0.12–2.78]	0.50
Thrombosis	6 (2.9%)	3 (2.8%)	3 (2.9%)	1.06 [0.23–4.76]	0.90
Obstruction of the device	16 (7.6%)	11 (10.8%)	5 (4.9%)	0.48 [0.17–1.39]	0.20

*VAS* Visual-Analogic Scale

## Discussion

Peripheral inserted central catheters and totally implantable vascular access ports are commonly used for CT treatment in patients with breast cancer [5, 6]. The use of PICC as an alternative to traditional chest-ports has increased over the last 2 decades, due to greater safety, ease of insertion and removal, and reduced waiting time [7]. Though, PICCs are more visible, they potentially may interfere with daily activities (e.g., bathing and showering) and are at a greater risk of dislodgement [8].

PICC-port is a particular type of arm TIVAP, inserted according to the current state-of-the-art of PICC insertion—ultrasound (US)-guided venipuncture of deep veins of the arm, micro-puncture kits, proper location of the tip preferably by intracavitary electrocardiogram (IC-ECG)—with placement of the reservoir at mid-arm [19]. The consistent adoption of these strategies has led to a considerable improvement in terms of safety and reliability of these devices, and recently, PICC-ports have been advocated as an alternative to chest TIVAPs to reduce complications and improve patients’ acceptance and QoL [28]. In fact, clinical studies reported positive clinical outcomes with the use of PICC-ports. In 418 adult patients with breast cancer undergoing chemotherapy [19], authors reported PICC-port failure rate of 2.6%, similar or even inferior to the same outcome reported for chest-ports in the recent literature [29–31]. Another more recent retrospective study [20] confirmed favorable clinical outcomes of PICC-ports in a very large cohort (4480) of cancer and non-cancer patients. In fact, over 80% of PICC-ports were removed because of the end of use, and severe adverse event (mainly infections requiring removal) occurred only in 1.1% of cases. Also, the rate of symptomatic thrombosis was 2%, requiring removal only in 0.02% of cases. Despite these encouraging clinical outcomes, PICC-ports’ QoL is not consistently considered among primary outcomes.

Breast cancer is the most frequent female tumor occurring in a relatively young and socially active population [1]; psychological/relationship needs and family/working habits represent unmet needs that must be considered [22]. Only one study [28] compared arm-ports to PICCs in terms of QoL in patients with colon and breast cancer. In their study, Burbridge and coworkers found that, despite pain scores associated with device implantation and device access for therapy were greater with ports compared to PICCs, overall, a significantly higher proportion of subjects with a PICC versus a port reported changes in the way they dressed, difficulty with showering or bathing, and having people comment on the presence of their device in the survey performed at baseline and at 3 months.

In our study, we found no differences between PICCs and PICC-ports in terms of pain at the insertion of the devices and in terms of discomfort associated with treatment. In particular, no significant differences were reported at the connection and disconnection of the infusion line to the two different devices, despite the positioning and removal of the Huber needle may be theoretically more painful [32].

On the other hand, in terms of QoL, PICC-ports were preferred over PICCs. By a greater extent compared to PICC-ports, patients carrying a PICC experienced the need to cover the device with clothing. In fact, patients may feel uncomfortable on showing their PICC to other people, as well as may feel anxious on possible dislodgement. Moreover, more patients with PICC were worried of the possibility of the device to be blocked. Also, a greater proportion of patients in the PICC group were bothered by the device when they shower, bathe, or perform personal hygiene or during social activities. In general, the degree of satisfaction was greater with PICC-ports. In fact, a significantly greater number of patients with PICC-port reported to be satisfied by the insertion of the device. Interestingly, those differences between PICC and PICC-port were even more pronounced in women under the age of 60. This is easily explainable by the fact that women of that age usually still work and are more socially active. Therefore, an external device such as a PICC may represent a limitation for their daily activities. Moreover, the more frequent (weekly) PICC’s maintenance compared to PICC-port (monthly or bimonthly) may make a difference for many women.

In terms of complications, a significantly greater incidence of ecchymosis, but not of hematoma, occurred in the PICC-port group, which is not surprising. As a matter of fact, PICC-port insertion, despite being less invasive than a chest-port, is a more invasive procedure than a PICC insertion [12]. Also, it must be considered that at the beginning, when we started inserting PICC-ports, pocketing the reservoir at the arm represented a technical challenge, which was easily overcome by practice. In fact, we progressively observed a reduction in the incidence of ecchymosis as the clinicians improved their technical skills. Nevertheless, ecchymosis, despite may alarm the patient, is a benign phenomenon, with a favorable outcome without any treatment.

Overall incidence of symptomatic catheter-related thrombosis (CRT) was low (3%), and no significant differences were found between the two groups, despite a significantly greater proportion of PICC-ports had a bigger diameter than PICC (5 fr vs 4 fr). This is basically due to the consistent adoption by our group of a CRT prevention protocol, both for PICC and PICC-port insertion, encompassing four strategies: (a) US-guided venipuncture, which limiting the number of attempts, reduces endothelial trauma and the consequent CRT risk; (b) attention to catheter-to-vein ratio, which, rather than catheter diameter per se, is one of the most important factors for determining the risk of CRT [33]; (b) the extensive use of catheter tunneling, which allows puncturing proximally to the axilla, where normally veins are larger; (d) accurate, intra-procedural tip location with intracavitary ECG [34]. As a matter of fact, recent meta-analysis demonstrated that, if those strategies are all consistently adopted, the risk of symptomatic PICC-associated CRT risk is very low and the risk of pulmonary embolism minimal or absent [35, 36].

Incidence of infection was very low. Only two cases of infection were recorded: one local (pocket) infection and one CRBSI—none of them requiring device removal—occurred in the PICC-port group, and none in the PICC group, with no statistical significance. This favorable outcome can be due to the scrupulous application of infection prevention strategies, as prescribed by the international guidelines [27, 37, 38].

This study has some limitations. First, it was carried out on patients with breast cancer undergoing neoadjuvant CT, and the results may not be representative of all patients with breast cancer. Hopefully, the role of PICC-ports in terms of QoL in other stages of breast cancer, and in other type of tumors, and in other healthcare systems (e.g., lower income countries) will be addressed by further research. Second, the experience with PICC-port implantation, especially at the beginning, was very scarce for the staff, but improved over time during the study period. This may have influenced the duration of the procedure and the incidence of complications. Third, some patients openly asked for a PICC-port to be implanted, and this may have influenced their attitude toward these devices.

In conclusion, in women with breast cancer undergoing neoadjuvant chemotherapy, PICC-port, compared to PICC, showed to interfere less with daily life activities, to be associated less with psychological and social concerns and to be associated with less anxiety for device being blocked or dislodged. In this population, and especially in youngers, PICC-port is overall better accepted than PICC in terms of QoL, with similar risks of complications.

## Data Availability

All data generated or analysed during this study are included in the published article.
